# Designing an Engineered Construct Gene Sensitive to Carbohydrate In-vitro and Candidate for Human Insulin Gene Therapy In-vivo

**DOI:** 10.22037/ijpr.2019.14650.12567

**Published:** 2019

**Authors:** Shivasadat Gheflat, Abdolrahim Sadeghi, Mojgan Bandehpour, Keyvan Ramezani, Bahram Kazemi

**Affiliations:** a *Cellular and Molecular Biology Research Center, Shahid Beheshti University of Medical Sciences, Tehran, Iran. *; b *Department of Biotechnology, School of Advanced Technologies in Medicine, Shahid Beheshti University of Medical Sciences, Tehran, Iran. *; c *Departement of Parasitology, School of Medicine, Shahid Beheshti University of Medical Sciences, Tehran, Iran.*

**Keywords:** Diabetes Mellitus, Homologous recombination, HeLa cell, LPK promote

## Abstract

Diabetes is a common disorder worldwide, and exhaustive efforts have been made to cure this disease. Gene therapy has been considered as a potential curative method that has had more stability in comparison with other pharmaceutical methods. However, the application of gene therapy as a definitive treatment demands further investigation. This study is aimed to prepare a suitable high- performance vector for gene therapy in diabetes mellitus. The designed vector has had prominent characteristics, such as directed replacement, which makes it a suitable method for treating or preventing other genetic disorders. The whole rDNA sequence of the human genome was scanned. The 800 bp two homology arms were digested by EcoRI, synthesized and cloned into the pGEM-B1 plasmid (prokaryotic moiety). The carbohydrate sensitive promoter, L-pyruvate kinase, and insulin gene were sub-cloned between homologous arms (eukaryotic moiety). The PGEM-B1 plasmid was digested by EcoRI, and the eukaryotic fragments were purified and transfected into Hela cell and then cultured. Afterward, the 300 µg/mL of glucose were added to the culture medium. Insulin expression in the transfected cells with 200 and 400 ng of the construct in comparison with negative control was detected using western blot and ELISA methods. Results have shown insulin expression in different glucose concentrates.

## Introduction

Diabetes is a disease, caused by abnormal metabolism of carbohydrates due to defects in insulin secretion, caused by pancreatic dysfunction or deficiency of insulin receptors on the cell surfaces. Insulin is a hormone secreted by pancreatic cells that stimulate liver cells to take up glucose from the blood and store it as glycogen in the liver to regulate blood glucose and glycogen accumulation in muscle cells as a source of energy boost. Lack of insulin secretion or deficiency in its function can cause several complications in the diabetic patient.

Given the importance of insulin in the regulation of metabolism (1), several methods have been tested to compensate for its lack. Gene therapy is a method that has been considered today as a one of the specific way for curation of several diseases. Transformation of the desired therapeutic gene into the cell genome is one of the most significant steps in gene therapy (2). The use of viral vectors because of their high capacity as well as the ability to insert into the host genome has been considered by researchers (3). Although the use of viral vectors for gene transfer into eukaryotic cells is a valuable phenomenon, this has had disadvantages that can affect the outcome of gene therapy. 

These disadvantages include reactivation of the virus (the vector) and simulation of host immune response. Due to failure of inaccurate insertion of the therapeutic gene into the genome (4, 5), researchers used non-viral vectors, including naked DNA (plasmid) to overcome this problem. The main advantages of non-viral vectors are‚ they have had safety function without stimulating of the immune system, cost-effectiveness, easy to use and have had a controllable expression in the cells. However, they have less efficiency than viral vectors (6, 7).

The goal of this study was to produce an engineered construction sensitive to carbohydrates in eukaryotic cells *in-vitro*. 

Accordingly, the human rDNA sequence which has many copies in the genome and is a useful target for gene transfer through homologous recombination (8) was used in our project.

## Experimental

*Construct design*

The homologous recombination process should have two conserved complementary homologous arms within target sequences (9). The eukaryote rDNA sequence has had many copies in the genome (8) and is a useful target for gene replacement. The whole rDNA sequence of the human genome (accession: U13369.1) was scanned and the selected area with a high score of 200 was highly conserved. The 800 bp two homology arms have been synthesized into pEX- A vector (concerning the position of restriction enzyme cutting sites for insulin gene and promoter sequence). 

The pEX-A plasmid was digested by EcoRI and the homologous arms sequence was released and sub-cloned into the pGEM-B1 plasmid (prokaryotic moiety). The carbohydrate (sugar) sensitive promoter, L-pyruvate kinase (181 bp) was used (10). 

The insulin gene (accession no AAA59172) and promoter were placed downstream of the promoter and were sub-cloned between homologous arms (eukaryotic moiety) into pGEM-B1 plasmid through BamHI and KpnI restriction sites. The vector was transformed into *E. coli*.

*Transfection*

pGEM-B1 plasmid was digested by EcoRI and, its eukaryotic moiety was released. The eukaryotic fragment was purified and transfected into Hela cells by electroporator (220V/ 500μs). 

For confirmation of the insertion gene into rDNA, two primers were designed: Forward primer at the homologous sequence and reverse primer on the insulin gene sequence that this primer design has had 860 PCR products.

Expression of insulin: Hela cells were cultured in RPMI1640 medium enriched by 10% FBS, 100 ug/mL penicillin and 100 µg/mL streptomycin (11, 12, 13). The 300 micrograms/ mL of glucose was added to the culture medium. Insulin expression in transfected cells with 200 and 400 ng of the construct, in comparison with negative control, was detected using western blot and Monobind ELISA kit (14).

## Results

*Construction of default vector*

**Figure 1:**
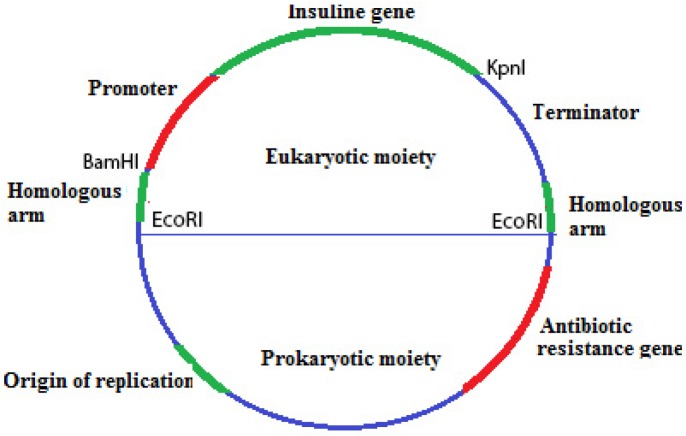
default vector which is made up of prokaryotic and eukaryotic sections

An overview of the construct has shown in Figure 1. Default vector has been made up from prokaryotic and eukaryotic moieties, for proliferation and laboratory experimental requirements.

*Designing of homologous arms*

The 800 bp two homology arms from rDNA sequence were synthesized into pEX-A vector (with concerning to the position of restriction enzyme cutting sites for insulin gene and promoter sequence) and subcloned into pGEM- B1 plasmid through EcoRI restriction enzyme. (Figiur 2)

*Cloning of insulin gene and promoter*

Insulin gene and promoter sequence has been ligated between two homologous arms through BamHI and KpnI restriction sites and confirmed by PCR reaction with designed primers on the two homologous arms sequence (Figure 3).

*HeLa cell culture and gene transfer*

Confirmed construct was digested by EcoRI restriction enzyme, and eukaryotic moiety was purified. HeLa cells were grown on, suspended cells were removed, and the purified construct was electroporated into the adherent cells. The transfected cells were confirmed by PCR as described in materials and methods. PCR Product (860 bp) produced by forward (insulin gene) and reverse (18srRNA) primers was electrophoresed on agarose gel (Figure 4).

*Insulin expression*

To stimulate transcription, 300 micrograms/mL of carbohydrate (glucose) was added to the culture medium (cell constructs were transfected, with 200 and 400 ng of the construct, respectively). Insulin gene expression was confirmed by ELISA (Figure 5) and western blotting (Figure 6). 

ELISA showed that in the negative control cells (no sugar) insulin was not expressed but, in sugar-stimulated cells, insulin was expressed in cell culture media.

**Table 1:** absorption of samples read by ELISA reader at 450 nm

**Samples**	**Absorption**
1: 5 μL (200 ng) from construct	0.248
2: 10 μL (400 ng) from construct	0.789
3: negative control	0.011

**Figure 2:**
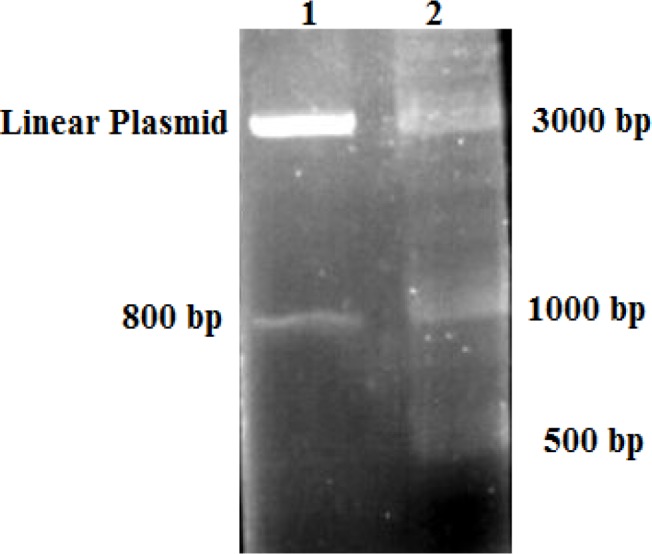
Identification of homologous arms (800bp) with EcoRI restriction enzyme. Lane 1: digested plasmid by EcoRI. Lane 2: I kb DNA ladder marker

**Figure 3:**
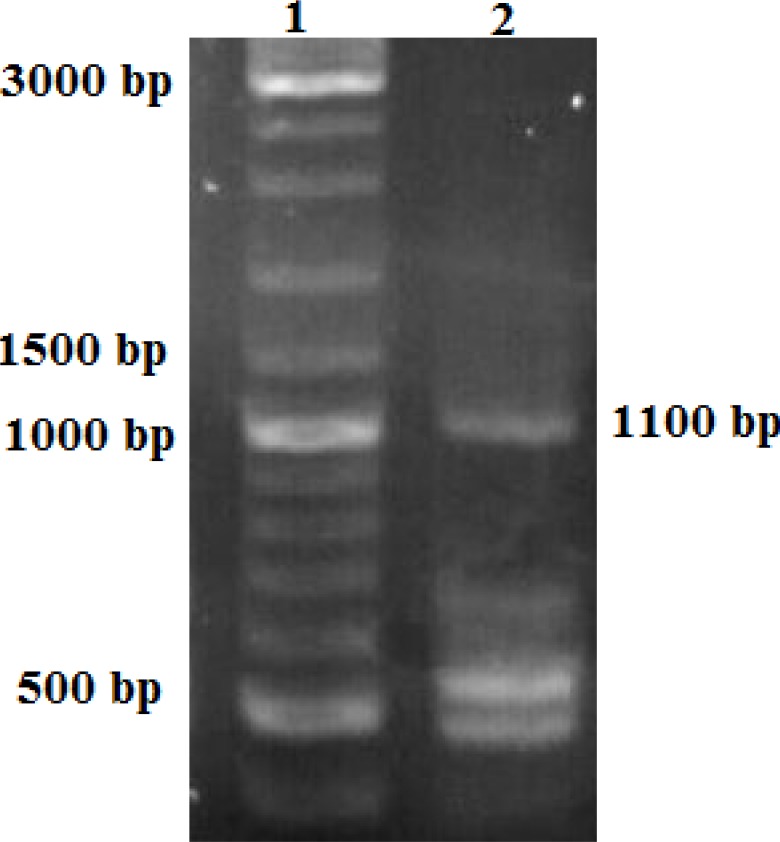
PCR products (110 0 bp) of confirmed whole construct include 511 bp insulin/promoter with 600 bp from homologous arms. Lane 1: 1 kb DNA ladder marker. Lane 2 PCR product on cells transfected by the eukaryotic moiety of default vector

**Figure 4:**
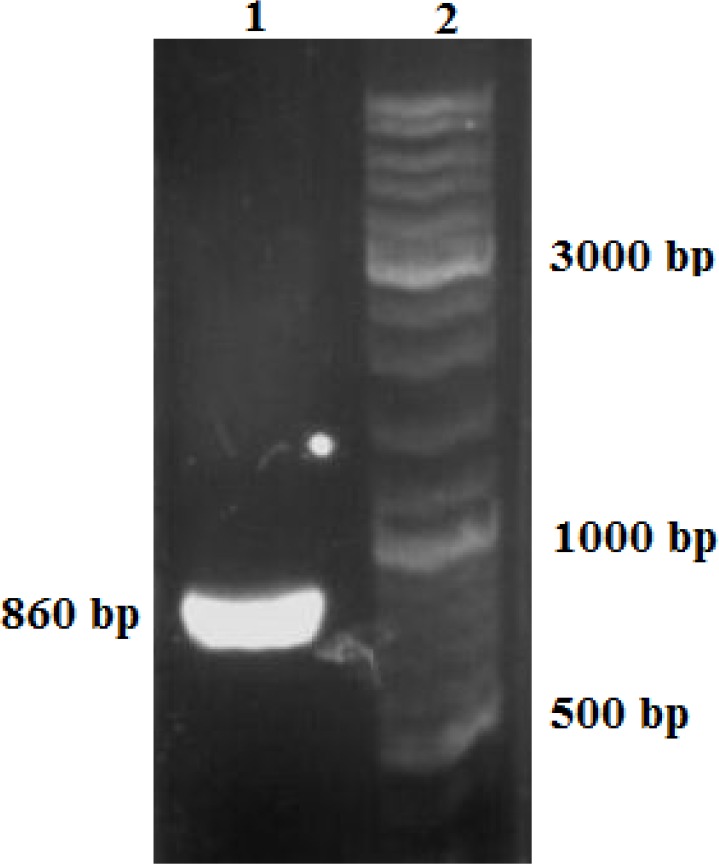
Confirmation of correct insertion in the genome in transfected cells Lane 1 PCR product of insulin gene and rRNA. Lane 2: I kb DNA ladder marker

**Figure 5:**
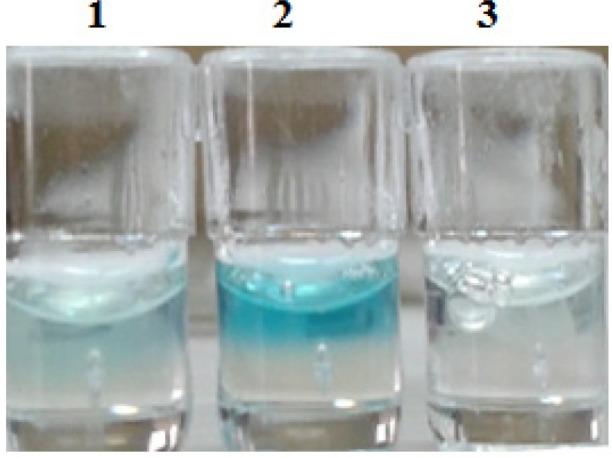
well 1: Hela cells were transfected by 200 ng of the construct, well 2: Hella cells were transfected by 400 ng of the construct, well 3: negative control (without carbohydrate)

**Figure 6:**
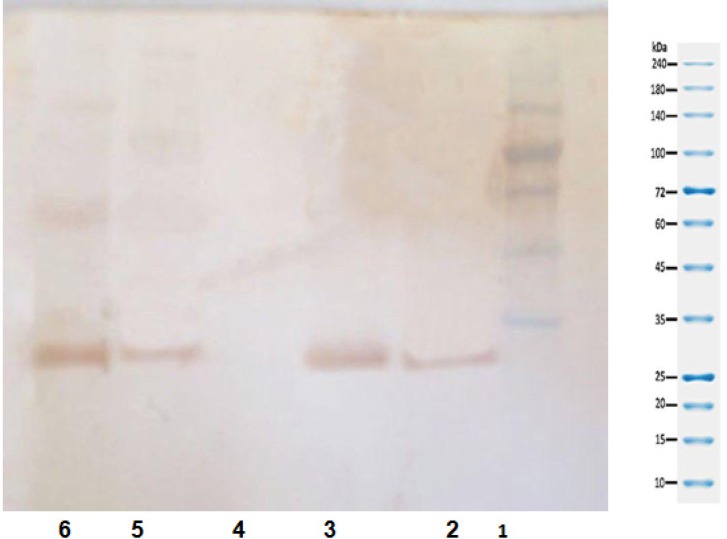
Confirmation of gene expression by western blot

Lane 1: protein marker.

Lane 2: sample replication and expression analysis of construct with added 400 ng of glucose.

Lane 3: A sample replication and expression analysis of construct with added 200 ng of glucose.

Lane 4: negative control without the addition of glucose.

## Discussion

Given the importance of diabetes and its treatment, in the current study, a plasmid construct for expressing the human insulin gene has been successfully designed and cloned. Our results have indicated that the construct has been capable of accurate targeting and gene transfer into the chromosome. The Specific carbohydrate sensitive promoter (10) has been used for insulin gene expression with the comparative advantage of providing several restriction enzyme recognition sites for replacing other genes into the same construct. Muzzin *et al*. treated diabetic rats by a recombinant adenovirus contained mutated preproinsulin cDNA. Briefly, they alter the B-C junction, from Lys-Ser-Arg-Arg to Arg-Ser-Lys-Arg, which is the consensus sequence recognized and cleaved by furin, a liver protease (15). Chen *et al*. used glucose 6 phosphatase promoters for controlling insulin gene that have been cloned in adenovirus and transfected into hepatocyte rat liver; they measure insulin in culture media of rat blood by ELISA kit (16). Dong *et al* treated diabetic rat using engineered preproinsulin cDNA gene under control of elongation factor 1-(EF1-) promoter and transfected into the liver cell by adenovirus (17), but we have used complete insulin gene under control of carbohydrate sensitive liver protein kinase promoter. We have designed a plasmid with eukaryotic and prokaryotic moieties. 

Despite many efforts, gene therapy is still not a routine medical treatment and progress is less than expected (7). However, gene therapy has had great potential in the treatment of genetic and metabolic disorders. The Long-term success of gene therapy has been based on the controlled transfer of targeted therapeutic gene and precise replacing of the mutated gene (18). 

Homologous recombination appears that have fewer side effects, and transfer of the therapeutic gene technique is a promising approach for gene therapy (9). In the field of human ribosomal gene sequences, homologous recombination technique recently has become very important (21, 22). This sequence due to the high copy number in cells chromosome measures to be considered within the genome (8, 21 and 22). 

Conclusion: we designed a naked DNA vector and carbohydrate sensitive promoter for transfer of insulin gene into rDNA of the mammalian cells. According to our information, this is the first transfer system into mammalian cells by naked DNA vector. 
